# Possible Early Generation of Physiological Helical Flow Could Benefit the Triflo Trileaflet Heart Valve Prosthesis Compared to Bileaflet Valves

**DOI:** 10.3390/bioengineering7040158

**Published:** 2020-12-08

**Authors:** Ch. Bruecker, Qianhui Li

**Affiliations:** School of Mathematics, Computer Science and Engineering, City, University of London, Northampton Square, London EC1V 0HB, UK; qianhui.li@city.ac.uk

**Keywords:** physiological helical flow, mechanical heart valve prostheses, aorta, PIV, trileaflet

## Abstract

Background—Physiological helical flow in the ascending aorta has been well documented in the last two decades, accompanied by discussions on possible physiological benefits of such axial swirl. Recent 4D-MRI studies on healthy volunteers have found indications of early generation of helical flow, early in the systole and close to the valve plane. Objectives—Firstly, the aim of the study is to investigate the hypothesis of premature swirl existence in the ventricular outflow tract leading to helical flow in the valve plane, and second to investigate the possible impact of two different mechanical valve designs on the preservation of this early helical flow and its subsequent hemodynamic consequences. Methods—We use a pulse duplicator with an aortic arch and High-Speed Particle Image Velocimetry to document the flow evolution in the systolic cycle. The pulse-duplicator is modified with a swirl-generating insert to generate early helical flow in the valve plane. Special focus is paid to the interaction of such helical flow with different designs of mechanical prosthetic heart valves, comparing a classical bileaflet mechanical heart valve, the St. Jude Medical Regent valve (SJM Regent BMHV), with the Triflo trileaflet mechanical heart valve T2B version (Triflo TMHV). Results—When the swirl-generator is inserted, a vortex is generated in the core flow, demonstrating early helical flow in the valve plane, similar to the observations reported in the recent 4D-MRI study taken for comparison. For the Triflo trileaflet valve, the early helical flow is not obstructed in the central orifice, similar as in the case of the natural valve. Conservation of angular momentum leads to radial expansion of the core flow and flattening of the axial flow profile downstream in the arch. Furthermore, the early helical flow helps to overcome separation at the outer and inner curvature. In contrast, the two parallel leaflets for the bileaflet valve impose a flow straightener effect, annihilating the angular momentum, which has a negative impact on kinetic energy of the flow. Conclusion—The results imply better hemodynamics for the Triflo trileaflet valve based on hydrodynamic arguments under the discussed hypothesis. In addition, it makes the Triflo valve a better candidate for valve replacements in patients with a pathological generation of nonaxial velocity in the ventricle outflow tract.

## 1. Introduction

The flow structures in the aortic arch during the systolic flow pulse show many complex flow features such as vortices, helical streamlines, secondary flows, and retrograde flow regions, all of which considerably contribute to the volume-averaged shear load on the blood cells. When a replacement of the natural valve is required, the artificial prosthesis will affect the overall hemodynamics by the alteration of these flow features in terms of size, magnitude and temporal evolution. The more the design of the prostheses differs from the natural valve, the more such non-physiological changes are expected, which makes the improved design of mechanical heart valves (MHVs) an ongoing research subject of great interest for engineers and clinicians. Despite advancements in the development of bioprosthetic surgical valves and transcatheter valves, there is still a need of such durable valves in younger patient population [1].

To estimate the effect of non-physiological alterations by MHVs, detailed flow studies in simplified mock circuits or CFD simulations are used to obtain the unsteady, 3D flow field data (4D flow field), as illustrated in [2,3,4,5] for instance. Those tests are often made with simplified phantoms of the ventricle and the aorta (straight tube), and mostly apply uniform inflow conditions. As mentioned in Li et al. [6], it is obvious that the valve prosthesis must be designed not only from a hydraulics viewpoint in an isolated environment but also by including the vessel walls into the design and qualification process. This includes the boundary conditions upstream of the valve (diastolic flow in the left ventricle and muscle contraction action) as well as downstream (curved aorta walls, 3D bend). The aortic walls are affected by the complex flow features generated downstream of the valve, causing additional stress loads. It was shown by Verma and Siu [7], that the direction of the blood flow along the aortic wall has an important influence on the degree of stress, which was concluded from the common disease of an aortic dilatation in patients with bicuspid aortic valves. It has been reported that high fluctuations in the magnitude of the Wall-Shear Stress (WSS) can lead to severe heart disease [8,9] and affect the aortic wall after implanting artificial heart valves [6,10]. Guzzardi et al. [11] showed that elevated WSS was associated with degradation of the aortic wall. Therefore, they highlighted the importance of investigating whether high-velocity jets and high WSS is found in the aorta after implantation of the prosthesis. WSS measurements using micro-pillar sensors reported in Li et al. [6] clearly showed considerable differences in the peak oscillations values of WSS generated by different designs of mechanical heart valves (MHVs).

**Figure 1:**
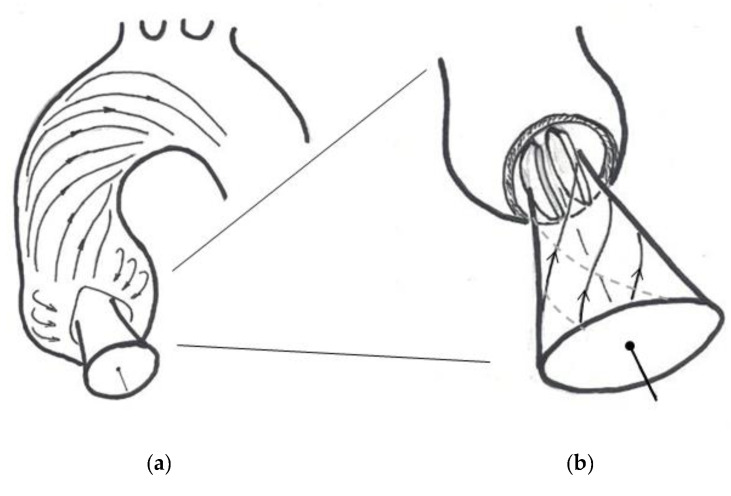
(**a**) Adapted sketch from the schematic drawing by Kilner et al. [18] to illustrate the evolution of helical flow in the aortic arch; (**b**) hypothesis of the authors with early helical flow generated in the ventricular outlet tract, illustrated by an artistic schematic drawing of a streamtube entering the valve plane. The scene is shown for the case of interaction with an implanted bileaflet mechanical heart valves (MHV).

A hitherto little addressed aspect in flow studies of aortic valve prostheses is the interaction of the valve with possible helical flow features in the aortic arch. Such helical fluid motion is not only relevant for the aortic arch, seemingly it is a common feature in natural flow physiology. Baratchi et al. [12] concluded that the role of helical flow in vessel physiology and pathology requires further study. De Nisco et al. [13] reported an inverse correlation between helical flow and the development of atherosclerotic disease, with one example of areas susceptible for atherosclerosis being the inner curvature of the aortic arch. The existence of a right-handed helical flow structure in the ascending aorta is now a well-acknowledged physiological flow feature [14]. This observation was made possible by the increasing quality of in-vivo velocity measurements such as phase-contrast magnetic resonance imaging (PC-MRI) [15,16,17]. It goes back to the MRI-study by Kilner et al. [18] of the velocity maps in transaortic planes (see Figure 1a), in which they concluded that the only reasonable interpretation of the combinations of axial and nonaxial velocity patterns is a right-handed helical flow in the upper arch.

More recent high-resolution 4D MRI studies reported in [19] indicate that helical flow usually starts already in early-systole in healthy volunteers. Similar, von Spiczak et al. [14] found a right-handed vortex arising in the ascending aorta early in systole and propagating to the aortic arch, with peak vorticity values reached in mid systole. As more and more 4D MRI data show a right-handed swirling flow early in systole, also directly distal to the valve, this flow feature probably is not only induced by the downstream curvature of the arch. The hypothesis introduced herein and illustrated in Figure 1b is as follows: the right-handed helix in the ascending aorta may partly being fed from early swirl in the ventricle outflow tract entering the aortic arch. The following arguments led us to this hypothesis:

- The convective nature of the swirl growth—The recent 4D-MRI studies in [14,19] showed that the swirl propagates from the valve plane further into the aortic arch as the systolic cycle progresses.
- Angular momentum generated during the filling procedure of the left ventricle, which does not completely cease until the beginning of the systole—It is documented in previous research that a vortex ring or a tumble vortex [20] is generated in the filling phase. The flow in the ventricular outflow tract, therefore, has a non-axial component.
- The helical ventricle contraction—It is reasonable to assume that the left ventricle contraction re-orients the spin-vector of existing angular momentum into the ejection direction, causing a swirl component of the axial flow. This is supported eventually by the left ventricle torsional motion during contraction and the helical myofiber architecture of the LV wall [21,22].
- The bathtub vortex effect—Any weak swirl in the convergent outflow tract is strengthened when the flow accelerates towards the valve plane. This is due to conservation of angular momentum, similar to a bathtub vortex when draining through a small orifice. In their original paper, Kilner et al. [18] also mentioned that the existence of a possible helical flow in the ventricular outflow tract could not be excluded; a definite conclusion was not possible because of the limits of resolution power of MRI.

Our study aims are two-fold: Firstly, we introduce a modified in-vitro pulse-duplicator which generates early helical flow in the valve plane. This allows us to compare the evolution of axial swirl in the ascending aorta over the systolic cycle and to compare the data with the most recent data from 4D-MRI, addressing the arguments of the hypothesis. Secondly, we want to understand the interaction of early helical flow with different designs of mechanical heart valve prostheses and the hydrodynamic consequences thereof, an example being a bileaflet valve, as illustrated in the sketch in Figure 1b. To achieve high resolution in both space and time, High-Speed Particle-Image Velocimetry (HS-PIV) measurements are carried out to improve our understanding of unsteady and pulsatile flow phenomena linked to the interaction of the swirling flow with the valve.

## 2. Materials and Methods

### 2.1. Pulse Duplicator

**Figure 2:**
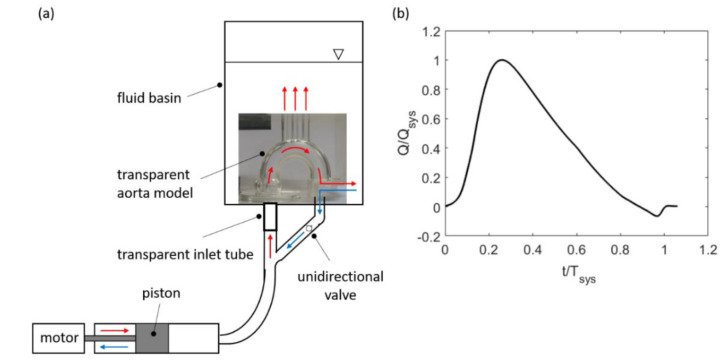
(**a**) Sketch of the pulse duplicator and (**b**) Flow profile in the systolic phase.

An in-house built pulse duplicator is used for the flow studies herein, a modified version of the one used in our previous study [6]. The core components are a programmable linear actuator with a piston at its axle, which is connected to a fluid tank that contains a transparent phantom model of an aortic arch (flat curved tube). The total pressure head can be adjusted by the air pressure within the tank, which plays the role of a compliance chamber to model systemic vascular compliance. Different flow resistances at the outlets can be adjusted by inserting nozzles into the openings of the phantom. A sketch of the experimental setup is shown in Figure 2a. The imposed flow profile for the current studies is given in Figure 2b, which shows a typical systolic flow pulse with maximum velocity reached at about 30% of the systolic cycle.

The systolic heartbeat with duration of *T_sys_* = 386 ms is characterised by an accelerated flow phase from 0 to 0.2*T_sys_*, then a peak systole phase from 0.2*T_sys_* to 0.4*T_sys_*, followed by a decelerated flow phase from 0.4*T_sys_* to 0.8*T_sys_*, and finally, the valve closure phase from 0.8*T_sys_* to *T_sys_*. At the peak of the flow, the peak inlet velocity reached a value of *U_P_* = 0.95 m/s. The mean velocity averaged over the complete beat cycle was *U_m_* = 0.19 m/s. The non-dimensional physical parameters of the flow can be represented by the Reynolds number Equation (1), Strouhal number Equation (2) and Womersley number Equation (3):

(1) $$Re_{P}=\frac{\rho U_{P}D_{A}}{µ},$$

(2) $$St=\frac{D_{A}}{2}\frac{f}{\left(U_{P}-U_{m}\right)},$$

(3) $$\alpha =\frac{D_{A}}{2}\sqrt{\frac{2\pi f\rho }{\mu }},$$

for a heartbeat rate f of 1.15 Hz (70 BPM), the Reynolds, Strouhal, and Womersley numbers were $Re_{P}=5340$, St=0.019 and α=16, respectively. These values of the non-dimensional physical parameters were similar to those that have been observed in the in-vivo experiment of the healthy aorta performed among 30 volunteers by Stalder et al. [23]. This indicates that the flow conditions used in the present study are representative for the natural flow situation.

The geometry of the aorta model was taken from Vasava et al. [24], who simplified the geometry derived from a three-dimensional reconstruction of a series of two-dimensional slices obtained in vivo using CT scan imaging on a human aorta by Shahcheraghi et al. [25]. The diameter of the aorta was *D_A_* = 25 mm, and the MHVs were then sized accordingly. At the arch, it has three branches in the form of three vertical tubes representing the brachiocephalic artery, the left common carotid artery and the left subclavian artery. The 180° curved bend representing the aortic arch with sinuses of Valsalva (SOV) has a planar-symmetry plane (crossing the left sinus in the middle), which eases the fabrication of the model. Further details of the model and the casting process were described in our previous work [6].

### 2.2. Heart Valve Prostheses

The effect of helical flow was studied for two different valve prostheses, one classical bileaflet mechanical heart valve, the St. Jude Medical Regent valve (SJM Regent BMHV) valve from (Abbott Laboratories, Chicago, IL, USA), and the Triflo trileaflet mechanical heart valve T2B version (Triflo TMHV) from Novostia SA, Neuchâtel, Switzerland, both size 25 mm (tissue annulus diameter) with an almost identical inner diameter (*D_A_* = 23.2 mm for the Triflo TMHV versus *D_A_* = 23.0 mm for the SJM Regent TMHV). The SJM Regent BHMV is a widely used MHV, which was introduced to the market in 1978. The Triflo TMHV consists of three leaflets that open to form a central orifice for the blood to flow through and three side orifices jets. The Triflo TMHV has been studied over the years in vitro [26,27,28], and its in-vivo performance has successfully been evaluated in animal studies [29]. As the design more closely resembles the natural aortic valves, it is assumed to have a better hemodynamic performance. A tubular insert is also used to mimic an open valve without any leaflets to obtain the “undisturbed” flow situation in the aortic valve plane. Those data were taken as reference.

**Figure 3:**
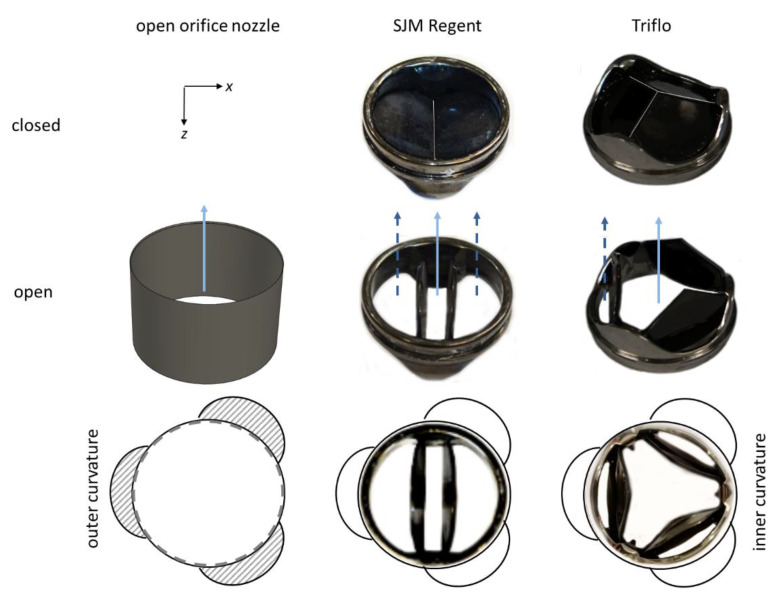
Tested heart-valve prostheses and their orientation within the phantom aorta. Left column: A tubular insert in form of an open orifice nozzle with circular cross-section and thin wall to cover the sinuses, forming a continuous, smooth tubular bend. Middle column: SJM Regent BMHV size 25 mm, with the leaflets in a fully open position (opening angle: 85°) and in the closed position (closed angle 25°). Right column: Triflo TMHV size 25 mm with the leaflets in the fully open position (opening angle 90°) and in the closed position (closed angle 40°). The solid arrow marks the centre orifice jet, and the dashed arrows mark the side orifice jets.

The arrangement of the heart valves is as displayed by the top view as shown in Figure 3, including the reference case, i.e., the tubular insert configuration, SJM Regent BMHV configuration and Triflo TMHV configuration. The thin-walled circular orifice nozzle with an outer diameter of 25 mm and an inner diameter of 24.6 mm was inserted into the annular valve plane, which covered the SOV, allowing the bulk flow to smoothly enter through the step-free inlet into the tubular bend. The BMHV and the Triflo TMHV were orientated, in both cases, such that one leaflet was facing the sinus at the outer curvature of the ascending aorta. For the Triflo TMHV, the other leaflets then faced the identical direction of the other sinuses. For the BMHV, the other leaflet was in the middle between two sinuses, facing to the inner curvature of the vessel wall.

### 2.3. Optical Flow Mapping

**Figure 4:**
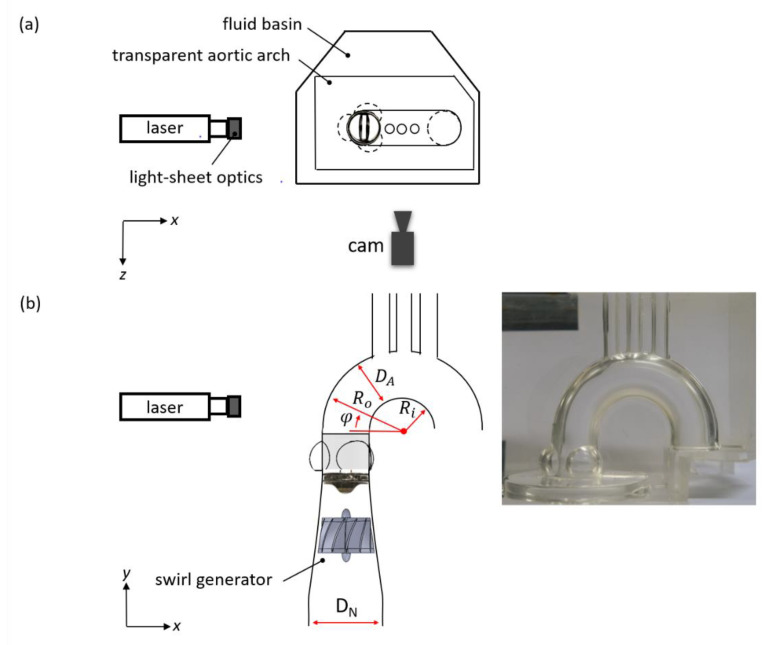
Details of the phantom aorta (including sinuses and brachiocephalic arteries) (*D_A_* = 25 mm, *R_i_* = 18 mm, *R_o_* = 43 mm) with additional inlet swirl generator module upstream of the aortic valve plane, (**a**) top view, (**b**) front view. The converging inlet nozzle had an initial diameter *D_N_* = 40 mm and length of 2*D_N_*, in which the swirl generator with an exit angle of 30° was inserted. For optical flow diagnostics, the transparent model was placed in a liquid container filled with the working fluid. This allows unobstructed view into the inner flow and velocity measurements using the HS-PIV method. The measurement plane can be arranged in the horizontal cross-section above the valve plane and in the vertical symmetry-plane of the bend.

The transparent phantom aorta was then placed at the bottom of the transparent basin (width/depth: 190 mm, height: 500 mm), as shown in Figure 4, and subsequently filled with a glycerine–water solution (58/42% by mass, density *ρ* = 1140 kg·m^−3^, dynamic viscosity *µ* = 5 mPa·s at the temperature of 38 °C, refractive index 1.41). The refractive index of the glycerine–water matches that of the silicone model such that there was undisturbed optical access into the inner of the aorta. It must be noted here that the working fluid in the present study followed a Newtonian behaviour, which differs from the non-Newtonian behaviour of blood.

### 2.4. High-Speed Particle Image Velocimetry

The HS-PIV system was the same as that used in our previous study [6]. Small tracer particles (fluorescent microshperes, mean diameter $d_{p}=$ 30 µm, Dantec Dynamics, density *ρ_p_* = 1040 kg·m^−3^) were added to the working liquid for the HS-PIV measurements. The Stokes number for these particles was $Stk=\tau U_{P}/d_{p}\approx 0.33\ll 1$, where the peak inlet velocity (*U_P_* = 0.95 m/s) was taken as the velocity scaler and the characteristic time scale was $\tau =\rho_{p\;}d_{p}^{2}/\left(18\mu \right)\approx 10\;\mu s$ [30]. This indicated that the particles were able to follow the streamlines closely. A continuous wave Argon-Ion laser (Raypower 5000, 5 W power at λ = 532 nm, Dantec Dynamics) was used as an illumination source. The output laser beam was approximately 1.5 mm in diameter and was further expanded into a sheet illuminating the symmetry plane of the aorta. Full format recordings of the flow in the AAo were taken with a high-speed camera (Phantom Miro 310, Ametek, Wayne, NJ, USA, 576 × 768 px^2^ recording at 7200 fps) equipped with a lens (Tokima Macro f = 100 mm, F 2.8) giving a field of view of 57.6 × 76.8 mm². Furthermore, HS-PIV measurements were taken in the radial cross-section distal to the SOV with the camera looking from top. The axial vorticity was then calculated from the velocity field in this plane by taking the curl of the velocity vector field at each data point. The unit of vorticity is given as s^−1^.

## 3. Results

### 3.1. Outlet Flow with Swirl

**Figure 5:**
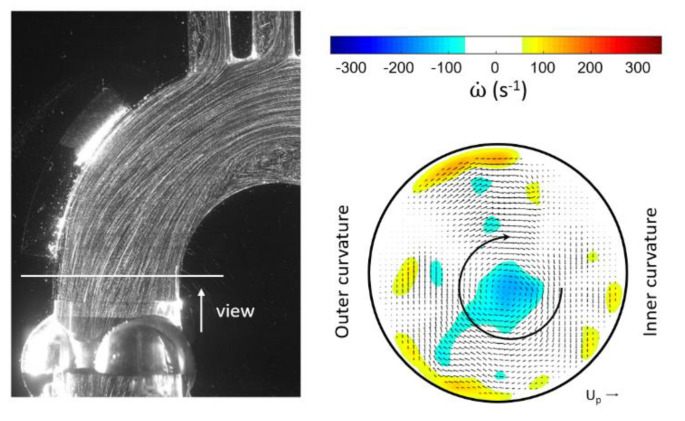
Circular orifice flow with inlet swirl at peak systole observed in the pulse duplicator. Left: Pathlines at peak systole shown by multi-exposure images in the light-sheet crossing the centre plane; right: velocity field in the cross-section distal to the sinuses of Valsalva (SOV) indicating the vortex core (color levels indicate axial vorticity, the inner circle shows the diameter of the vortex core). The length of the reference vector indicates the peak inlet velocity (*U_P_* = 0.95 m/s).

The hypothesis of generation of early helical flow in the valve plane has been investigated based on a comparison of our results with published data, the most relevant being the recent study from von Spiczak et al. [14] using high-resolution phase-constrast MRI on healthy volunteers. As we could not reproduce the data for a natural valve at a cross-section distal to the valve, we used the open orifice (see Figure 3 left) in the pulse-duplicator, which represents approximately a fully open natural valve. The opening phase is not considered herein. Citing their results for healthy volunteers [14] (p. 12): “clock-wise CW rotational flow is developed during early systole. It could be observed first in ROI 0 and 1, which were located directly above the aortic bulb and in the ascending aorta, respectively (ROI 0: minimal vorticity = −166.4 ± 86.4 s^−1^ at 12% of cardiac cycle and ROI 1: −183.6 ± 65.3 s^−1^ at 16%). Thereupon, rotational flow propagated to the more distal ROIs 2, 3, and 4 with minimum values seen in mid systole (ROI 2: −240.1 ± 45.2 s^−1^ at 16%, ROI 3: −229.2 ± 50.0 s^−1^ at 20%, and ROI 4: −201.6 ± 92.7 s^−1^ at 16%)”. In addition, as mentioned in [14] (p. 1): “strength, elongation, and radial expansion of 3D vortex cores escalated in early systole, reaching a peak in mid systole (strength = 241.2 ± 30.7 s^−1^ at 17%, elongation = 65.1 ± 34.6 mm at 18%, expansion = 80.1 ± 48.8 mm^2^ at 20%), before all three parameters similarly decreased to overall low values in diastole.” The HS-PIV measurements in the radial cross-section distal to the sinotubular junction correspond to their Region of Interest (ROI) ROI 1. The velocity data obtained in this plane in the experiments allow for quantification of the axial vorticity (the spin-vector of the helical flow is in axial flow direction). As in agreement with their definition, negative values of axial vorticity stand for clockwise (CW) fluid motion. This direction is in the same direction as the physiological helix. Figure 5 shows the experimentally obtained contours of the axial vorticity overlaid on the cross-sectional vector field. The core of the CW vortex can be observed by the region of concentrated negative vorticity (in blue). An indication of the diameter is given by the circular arc, which is drawn along the radius of maximum tangential velocity (maximum value of about 0.5 m/s). The diameter of the arc is about 10 mm, which yields a core “expansion” of 75 mm^2^, well in the range reported by von Spiczak et al. [14]. Note that the core centre is slightly offset from the geometrical centre, which might be a result of the upstream effect of the arch.

An important benefit of the HS-PIV recordings is the potential to use the images for creation of a flow visualization picture showing segments of the flow path lines. The flow path lines displayed in Figure 5 highlight the difficulty in capturing the helical character of the flow from only this information in a planar cross-section. What looks like a rather parallel flow with little tilt towards the inner curvature is the result of the laser-sheet plane cutting the centre of the vortex core in the longitudinal plane. As the visualization shows only projections of short segments of the 3D particle paths, the circumferential motion is not readily visible, only it would be when the laser-sheet plane moved out of the centre to the edge of the vortex core. This is where considerable circumferential velocity (max 0.5 m/s) of 50% of the peak axial velocity exists simultaneously. The edge of the core is indicated in Figure 5 as a circular ring in the radial cross-section, where the maximum circumferential velocity appears. This observation also supports the methodology of a rigorous quantitative approach from the MRI data as done by von Spiczak et al. [14] to extract fundamental quantities such as vortex core size, strength and temporal evolution of those quantities in different planes and regions of interest (ROI).

**Figure 6:**
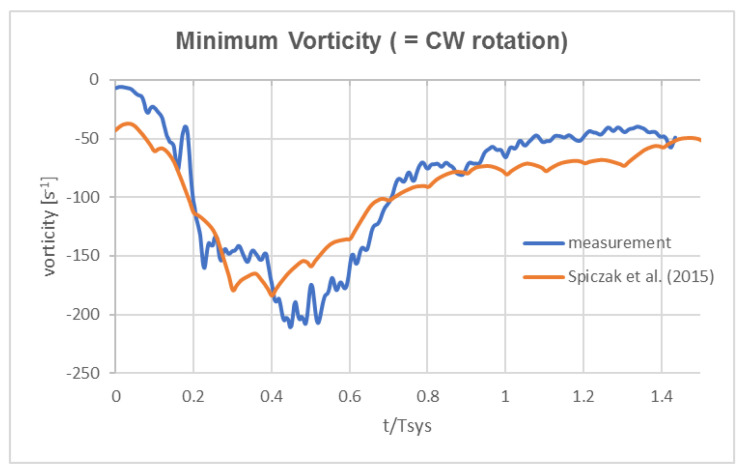
Peak negative vorticity value in the vortex core at the cross-section distal to the SOV in the Pulse Duplicator compared to the 4D MRI flow data of von Spiczak et al. [14] in ROI 1 at a similar position. Note the early development of the vortex in the plane distal to the SOV.

Additional information is given by the temporal evolution of the peak negative value of axial vorticity in the core. The experimental data were again compared to the values from von Spiczak et al. [14], as shown in Figure 6. Both plots show a similar profile with early generation of a core region of concentrated axial vorticity. Peak minimum values were reached about mid systole between 0.4–0.5*T_sys_*. The similar trend in the temporal evolution near to the valve plane hints that there is reasonable support to our hypothesis that early swirl could have been generated in the ventricular outflow tract and been transported into the ascending aorta. Note that possible swirl in the ventricular outflow tract may have been overlooked as it is only intensified when accelerated towards the valve orifice, similar to a bathtub vortex when draining into a small orifice (due to conservation of angular momentum in a convergent flow tube).

### 3.2. Helical Flow Interaction with MHVs

HS-PIV was used to study the influence of early helical flow on the hydrodynamics of MHVs in the ascending aorta, comparing the Triflo TMHV with the SJM Regent BMHV. For better visibility of the effect of early helical flow, we compared the situation with/without swirl generator inserted. The velocity fields in the centre plane (symmetry plane of the phantom) are shown for three selected instants in the peak systole, first as color-coded contours of constant axial velocity and secondly, as profiles of the same component of the velocity vector at different angles of the arch.

**Figure 7:**
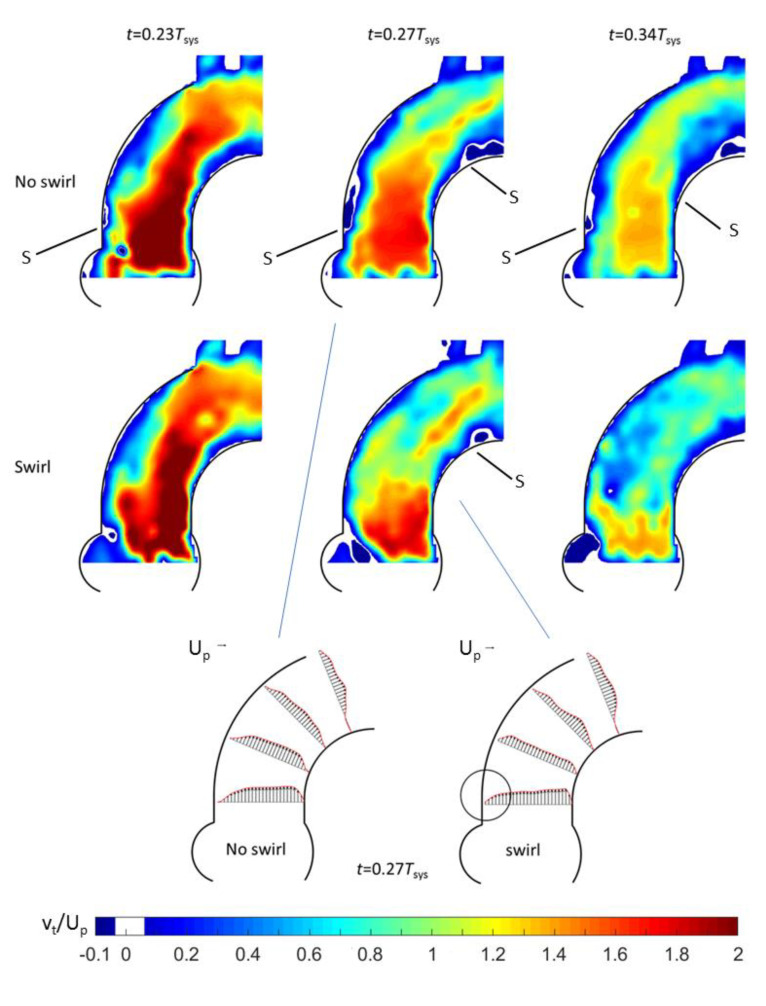
HS-PIV visualization of the flow evolution in the centre plane of the phantom aorta for the Triflo valve with/without inlet swirl in the Pulse Duplicator: Color-coded contours of constant axial velocity (top) and axial velocity profiles in the centre plane. “S” indicates a separation point.

For the Triflo TMHV (see Figure 7), in the early systole, the overall flow pattern in the centre-plane in the arch was not greatly affected by the presence of the swirl. Later in the systole, a difference was clearly visible in the lateral boundary of the core jet. For the no-swirl situation, the core penetrated straight into the aorta and flow separation was observed at the outer and inner curvature wall further downstream. For the swirl-case, the core had angular momentum, which resulted in a radial pressure gradient (pressure minimum at the centre). When the central jet left the valve region, it immediately spread radially and the flow attached to both the inner and the outer curvature wall. Consequently, the core widened and the velocity profile plateaued in the central region. The radial spread also re-energized the flow in the near-wall regions, which was demonstrated by the increased axial velocities near the vessel walls compared to the no-swirl case (marked as the circular region in Figure 7). Therefore, the boundary layer flow in the swirl case was more resistant to adverse pressure gradients, thereby overcoming the risk of flow separation. Indeed, the swirl case only had a marginal separation at the inner vessel wall compared to the no-swirl case. Another observation was a much stronger recirculation within the sinuses for the swirl-case. This supports an early closure onset of the leaflets of the Triflo TMHV at 0.77*T_sys_* compared to 0.8*T_sys_* in the no-swirl case (no early helical flow).

**Figure 8:**
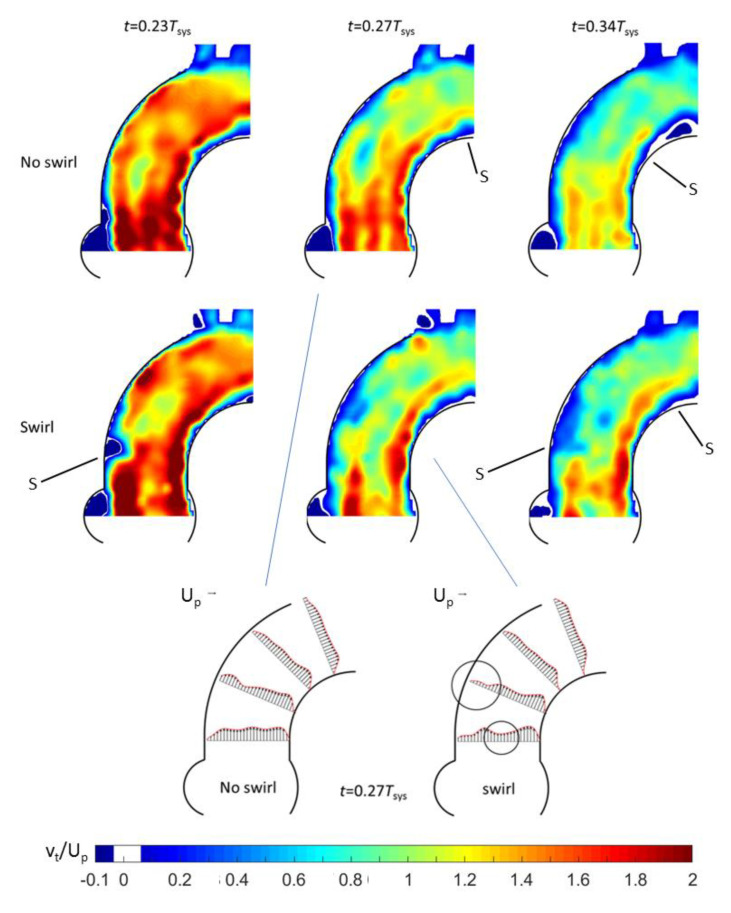
HS-PIV visualization of the contours of axial velocity magnitude (top) and axial velocity profiles in the centre plane during systole in the Pulse Duplicator for BMHV. “S” indicates a separation point.

Results for the BMHV are given in Figure 8. As for the Triflo TMHV, in the early systole, the flow in the centre-plane in the arch was not greatly affected by the presence of the swirl. For the no-swirl situation, one can see a clear indication of the three ejection jets, the two side-orifice jets and the core jet between the leaflets at the centre of the valve. A striking effect of the swirl was the drastic reduction in the velocity in the central jet. This reduction in the axial velocity can be explained by the existence of a concentrated streamwise vorticity in the core when it was interacting with the divergent flow channel formed between both leaflets. The flow passage between the two leaflets was comparable to a swirling flow through a diffusor with an opening angle of 5–10°. As known from the phenomenon of vortex breakdown, this causes a reduction in the axial velocity predominantly at the centre, sometimes even leading to flow reversal along the swirl axis [31,32]. Due to the velocity deficit in the centre, more flow was diverted into the side-orifice jets, as seen by the larger axial velocities therein. Both jets attach distal to the vessel walls, locally increasing the WSS values. While the swirl had a positive effect on the retarded region at the inner curvature of the aorta, it had a negative effect on the flow along the outer curvature as the large blue-colored region of near-stagnant flow grew in size along the curve.

## 4. Discussion

**Figure 9:**
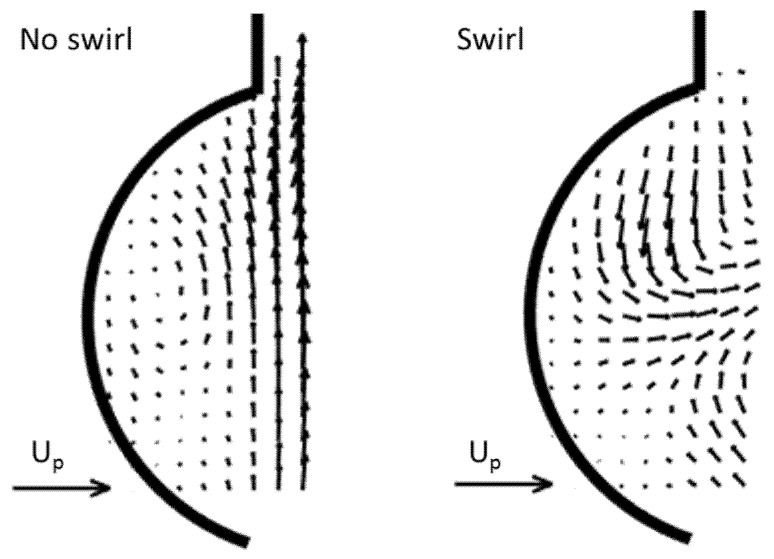
Enlarged view of the velocity vectors in the left sinus for the TMHV without and with swirl (t/T_sys_ = 0.34) with radial inwards-directed flow supporting early valve closure (with swirl).

The present work investigates, for the first time, the influence of early generation of helical flow in the valve plane on the hydrodynamics of different types of mechanical heart valve prostheses. Although the phantom aortic arch is curved only in one plane, we could see beneficial effects of the early helical flow in reducing or preventing retrograde flow at the inner curvature of the arch. As the Triflo TMHV allowed the swirling core flow to convect through the valve pane and further downstream without objecting the tangential velocity, angular momentum in the flow was conserved. Downstream of the valve, the radial pressure gradient in the core led to a radial expansion of the core region over the complete radial cross-section of the ascending aorta. This reduced the peak velocities in the core jet and the shear-stresses in the shear layer between the core jets and the annular region near the vessel wall. In addition, stronger retrograde flow was found in the sinuses, supporting the early onset of leaflet motion in the closing phase at 0.77*T_sys_* in the swirl case compared to 0.8*T_sys_* in the no-swirl case (no early helical flow). Figure 9 shows a magnification of the flow in the left sinus, which is located at the outer curvature of the arch. The enlarged region in the sinus illustrates stronger backflow vectors and a radially inwards-directed flow for the swirl-case, which supports the closure phase of the corresponding leaflet.

The impact of early helical flow in the valve plane on the flow distal to the BMHV was seen by a change in flow-partitioning between the three jets—the two side orifice jets and the centre jet. The latter shows reduced velocity in the centre while the side orifice jets became stronger; therefore, the valve guides a higher amount of fluid through the side orifices compared to the centre.

The bileaflet valve with its leaflets oriented in the axial direction works, in principle, as a flow-straightener by objecting the nonaxial flow components (see Figure 1b). The nearly parallel planes of both leaflets guide the flow in the axial direction, similarly to the guide-vanes of a stator in turbomachinery. Such a device is usually implemented at the exit of a flow pump to convert kinetic energy of the swirling flow into higher pressure. This is achieved by reducing the tangential velocity direction downstream to zero gradually using curved vanes. However, a larger energy loss (total pressure loss) occurs if the guide-vanes are planar and parallel to the axial flow, similarly to the leaflets in the BMHV. Therefore, under the conditions of early generation of helical flow entering the valve plane, this effect causes an additional hydrodynamic pressure drop in the BMHV that the left ventricle must overcome to eject the same amount fluid into the systolic pulse compared to the natural valve. This is contrary to the herein described Triflo TMHV, which has a design where the leaflets open the central area without obstructing bodies. Therefore, the core of the swirl flow can penetrate through the valve while conserving angular momentum.

**Figure 10:**
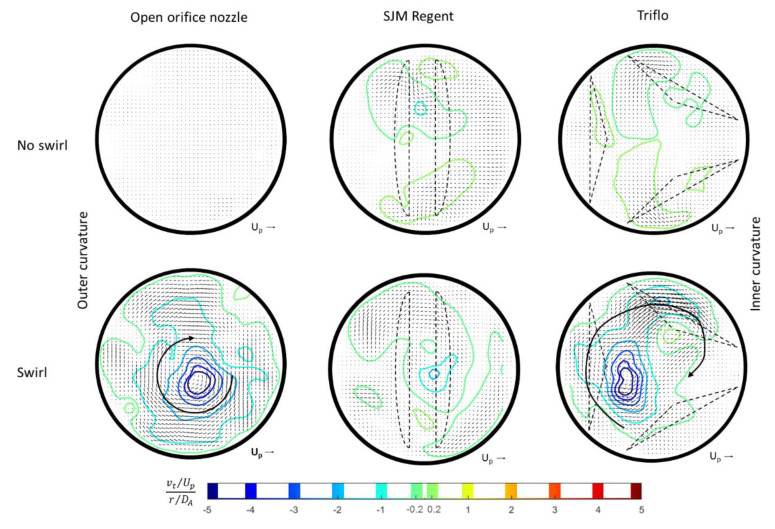
Cross-sectional flow field distal MHVs with contours of regions of constant angular velocity around a common centre of rotation. As a reference, the flow field for the open orifice is included at the left of the figure, too. The flow field is averaged over the peak systolic phase.

Figure 10 illustrates the angular momentum distal to the two MHVs. The HS-PIV measurement sequence in the radial cross-section was averaged over the peak-systolic phase with near-constant flow to reduce noise. The plots display the averaged velocity vector field in the cross-section (quiver plot) overlaid with contours of constant angular velocity around a common centre.

The area enclosed by the contour lines represents the fluid portion rotating in circumferential motion around the centre in a coherent motion. For this display, the velocity vectors were split into their tangential and radial components (relative to the centre) and only the tangential one was used to determine the angular velocity by dividing it with the radial distance to the centre. For better visibility of the difference between the no-swirl and swirl-cases, both results are presented in Figure 10 for each MHV. Note, that the no-swirl case should not show any large fluid parcels rotating round the geometrical centre, while local patches of higher vorticity may exist in regions of vortices generated by the leaflets. In general for no-swirl it is seen that fluid parcels rotating clockwise counter-balance with parcels rotating counter-clockwise. With imposed swirl, the results demonstrate that distal to the BMHV, most of the angular momentum is lost. In comparison, the core region in the TMHV shows a clear dominance of clock-wise swirl, similar in area and peak values to the open orifice case. The centre is somewhat offset to the left away from the geometrical centre, which is due to the upstream effect of the arch. As a side note, for the open orifice, there is a difference in the vorticity in Figure 5 compared to the constant angular velocity about a common centre of rotation, with the latter showing the coherent circumferential motion much clearer. A quantitative analysis of the mean angular velocity averaged over the cross-sectional area shows about 80% of the angular momentum was conserved with the TMHV compared to the open orifice—all concentrated in a coherent swirling motion. In contrast, only 30% survived when flowing through the BMHV in an irregular manner. Thus, the helical flow is conserved with the TMHV.

## 5. Conclusions

The present study investigated the hypothesis of early generation of helical flow in the valve plane of the aortic arch and its consequences on the hydrodynamics of MHVs and the flow in the arch. Firstly, we see the hypothesis supported by the following observations:

- Good agreement of the measured axial vortex in the valve plane of the aortic arch in the Pulse-Duplicator with recent published data of 4D MRI studies in the aortic arch of healthy volunteers.
- In the Pulse Duplicator, the axial vortex was generated with a swirl-generator inserted into the inlet tube upstream of the valve while in the MRI data, it was a result of the complex flow evolution during left ventricular ejection into the aortic arch. The near overlap of the magnitude and temporal evolution data between our experiment and the MRI suggest that early generation of helical flow in the valve plane indeed could originate from swirl in the ventricular outflow tract, which is convected into the arch.
- As mentioned already by Kilner et al. [18], the tortuous, S-shaped form of the vertebrate heart causes multidirectional intracardiac flow patterns, which may cause efficient directional exchange of energy between muscle and blood in the ejection. From reconstructions of path lines patterns alone, it is difficult to detect such a helical motion in the valve plane. Rather, quantifying the regions of concentrated axial vorticity in the cross-sectional planes of the ventricle and arch is necessary, similar to what has been done herein for the HS-PIV measurements and by von Spiczak et al. [14] in their MRI data.

Secondly, with the above given reasonable arguments of the existence of early generation of helical flow in the valve plane, the hydrodynamics of MHVs requires a closer look with regard to this effect on different aspects of the valve design. With existence of early generation of helical flow in the valve plane, the following conclusions can be drawn based on the results and hydrodynamic arguments:

Conservation of angular momentum for the Triflo TMHV—It preserves angular momentum in the core flow, which helps to re-distribute and expand the core flow distal to the valve towards the vessel walls of the aortic arch, reducing the potential of flow separation in the inner and outer curvature of the arch. Therefore, the physiological helical flow is preserved throughout the valve plane and the aortic arch. This might also have consequences on the distribution of existing micro-bubbles (MB) in the flow. It has been reported in literature that MB can be generated at mitral MHV due to degassing during the localized pressure drop at valve closure, see [33]. Their existence has been proven by the detection of high-intensity transient signals (HITS) in the echo of Doppler echocardiography at the instant of valve closure. Other sources for the generation and transport of MB into the left ventricle could be by left ventricle assist devices (LVAD). If those MB have a longer life-time, they could then be transported from the left ventricle via the aorta into the carotid arteries, causing gas embolism, including ischemia and strokes [33]. In general, MB tend to migrate to the region of minimum pressure, which for swirling flows, is the core of the vortex. Consequently, when physiological helical flow is preserved while passing the TMHV valve, micro-bubbles entering the aorta remain in the centre of the helical flow and, therefore, there is less risk to enter the branches of the brachiocephalic artery and carotid arteries.

Flow straightener effect for the bileaflet BMHV—Flow partitioning between the side-orifice and centre orifice ejection jets changes due to the complex interaction of the swirling flow with the leaflets, which act as guide vanes. The axially oriented parallel leaflets obstruct nonaxial flow components; therefore, the conversion from helical flow to axial flow does not happen in a smooth, loss-free way within the vane channels. There is a consequent drop in kinetic energy (total pressure loss) of the flow when passing the valve. Therefore, the left ventricle afterload for fluid ejection through the bileaflet valve is expected to be higher than for the Triflo TMHV, as the helical flow is preserved in the latter. The impact of the ejection jets on the aortic wall (in direction and magnitude) is predefined by the different designs of the two types of valves (TMHV versus BMHV). Therefore, the pre-stressed viscoelastic behavior of the aorta will respond in a different way to those valves (see Tagiltsev and Shutov [34]), which may also affect the degree of long-term damage. It is assumed herein, that early physiological helical flow and conversation of angular momentum through the TMHV helps to distribute the jet impact load in a random manner from cycle to cycle along the circumference of the aortic wall, other than in BMHV where the jet impact location remains constant and is therefore causing permanent punctual loads at the same location.

Overall, the observations under the given hypothesis provide more reasons for a superior hemodynamics of the Triflo TMHV against bileaflet valves on its own. Further studies are planned with the latest version of the Triflo valve in the Pulse Duplicator and in 4D-MRI studies of an isolated pig-heart. In the latter, more detailed information of the flow in the ventricular outflow tract will be obtained to test the hypothesis under realistic geometrical and physiological conditions of a beating heart. In addition, the experiments in the Pulse-Duplicator will be repeated with an in-house developed Scanning PIV technique to obtain the 3D volumetric velocity field instantaneously in the whole arch.
